# Effects of potato and sweet potato flour addition on properties of wheat flour and dough, and bread quality

**DOI:** 10.1002/fsn3.2693

**Published:** 2022-01-21

**Authors:** Hongwei Meng, Chong Xu, Meiying Wu, Ying Feng

**Affiliations:** ^1^ Food College Shenyang Agricultural University Shenyang China

## Abstract

The effects of 10%–30% of wheat flour substitution with potato flour (PF) and sweet potato flour (SPF) on the flour and dough properties, the total polyphenol (TPC), and carotenoid contents (TCC) of bread, as well as their correlation with bread texture and starch digestibility, were investigated. With PF and SPF addition, the peak, breakdown, and setback viscosity of the flour decreased. The addition of PF and SPF reduced the dough formation and stabilization duration, as well as the hardness of the bread. The specific volume of the bread depended on the addition amount of PF and SPF. When the addition of PF and SPF was 15%, the bread had the lowest hardness and highest specific volume. The TPC and TCC in the bread depended on the added flour variety, and negatively influenced specific volume and positively influenced the content of resistant starch (RS).

## Graphical abstract

The decreased extent of the peak, breakdown, setback viscosity of the mixed flour depended on the flour variety. Specific volume of bread depended on the variety and amount of added flour. TPC and TCC negatively influenced specific volume, elasticity, and RDS content.

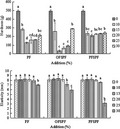

## 1 INTRODUCTION

Both potato and sweet potato are rich in starch, protein, vitamins, minerals, and fiber (Nzamwita et al., 2017; Wang et al., 2020; Zhang et al., 2017). Moreover, they contain bioactive phytochemicals such as polyphenols that exert antioxidant and anticancer effects (Liu et al., 2016; Zhu & Sun, 2019). Commercially, potatoes and sweet potatoes are mainly processed into starch, chips, and French fries (Tong et al., 2020; Zhang et al., 2017). In recent years, many countries have used potato and sweet potato as staple foods; therefore, their deep‐processed products, such as bread, mantou, and noodles, which satisfy the dietary habits of residents, are being studied and developed (Azeem et al., 2021; Liu et al., 2016; Zhang et al., 2017; Zhu & Sun, 2019). Because fresh potato and sweet potato are difficult to store, it is better to process them into flour, which not only maintains their nutritive composition effectively but also prolongs their supply time.

Studies have shown that replacing part of wheat flour (WF) with potato flour (PF) or sweet potato flour (SPF) not only fortifies its nutritional value but also improves the properties of the flour and dough, and the texture and sensory quality of the bread. Meng et al. (2020) replaced 15% of WF with PF to make bread, and found that the specific volume of the bread increased to 4.12 ml/g, the moisture content of the bread increased by 7%, and the hardness reduced by 6 N. Meanwhile, the color, flavor, volatile aroma, and overall acceptable levels of the bread increased significantly. Nzamwita et al. (2017) found that 20% and 30% substitution of orange‐fleshed sweet potato flour (OFSPF) for WF to make bread can combat vitamin A deficiency in developing countries. Azeem et al. (2021) found that the sweet potato‐wheat bread (SPWB) made from 45‐μm particle size of OFSPF exhibited higher specific volume and lower hardness. The network and viscoelasticity of its dough were also improved. Jia and Zhong (2018) found that the addition of the sweet potato residue obtained after the extraction of starch significantly influenced the water absorption of WF, the formation time, stability time, and the tensile force of the dough, as well as the hardness and sensory score of the bread. However, studies regarding the comparative effects of the addition of PF and SPF to WF on the characteristics of the mixed flour and the dough, and the texture, total polyphenol content (TPC), and total carotenoid content (TCC) of the bread are rare.

The aim of this study was to investigate the addition of PF and SPF, including OFSPF and purple‐fleshed sweet potato flour (PFSPF) on the characteristics of mixed flour and the responding dough, as well as the quality of bread, including texture, bioactive components, and digestive property. Based on this, the relationship between the TPC, TCC, and the texture and digestibility of bread was explored. Our study outcomes provide a theoretical and practical basis to produce potato‐wheat bread (PWB) and SPWB, and help accelerate the development of potato and sweet potato‐based resources in the staple food industry.

## 2 MATERIALS AND METHODS

### 2.1 Preparation of potato flour (PF) and sweet potato flour (SPF)

The commonly consumed Chinese varieties of fresh potato (eshu 10), OFSPF (Yanshu 25), and PFSPF (Ziluolan) were purchased from the market of Shenyang. The potatoes were washed, peeled, sliced, steamed for 20 min, and then dried using a heat pump dryer (CN‐HGJ12P‐Shanghai Guangzheng Medical Instrument Co. Ltd.) at 60°C for 12 h. Thereafter, they were crushed using a grinder (XM‐600‐Yongkang Boou Hardware Products Co. Ltd.) and passed through a 50‐mesh sieve, sealed, and stored at 4℃ for use.

### 2.2 Preparation of Sweet‐type bread

To prepare the bread, an egg, 15 g of butter (COFCO, China), 90 g of water, 40 g of white sugar (COFCO, China), 0.5 g of salt (China National Salt Industry Corporation), 200 g of mixed flour, 7 g of milk powder (Nestle S. A.), and 3 g of yeast (Angel Yeast Co., Ltd) were put into a bread maker (MM‐ESC1510, Midea). The WF (high gluten, COFCO) was replaced at ratios of 0%, 10%, 15%, 20%, 25%, and 30% with PF, OFSPF, and PFSPF to make the bread. The operation conditions of the bread maker were set as sweet bread, light color, and 750 g.

### 2.3 Measurement of the pasting characteristics of the flour

The pasting characteristics of the WF with different substitutions of PF, OFSPF, and PFSPF were measured using a rapid viscosimeter (RVA‐Super3, Newport Scientific Pty Ltd, Australia) using the methods described by Zhu and Sun (2019). Briefly, 3.5 g flour mixture was blended with 25 ml of deionized water, held at 50℃ for 5 min, heated to 95°C at 6°C/min, held at 95℃ for 5 min, and finally cooled to 50°C at the same speed and held at 50℃ for 2 min. The peak viscosity, through viscosity, breakdown viscosity, final viscosity, setback viscosity, and pasting temperature were measured from the viscograph.

### 2.4 Measurement of the thermomechanical characteristics of the dough

The thermomechanical characteristics of the dough were measured using the MixolabⅡenzyme rheometer (SYD‐0716, Chopin). Using the method described by Azeem et al. (2021), with some modifications, the weight of the dough was set at 200 g. The instrument automatically added the needed amount of water to make the mixed flour and form the dough in accordance with the requirement of achieving optimal consistency and maximum torque of 1.1. The stirring speed was set at 80 rpm and the temperature program was set as follows: the sample was held at 30℃ for 8 min, then heated to 90℃ at 4℃/min, and held for 7 min followed by cooling to 30℃ at the same speed.

### 2.5 Measurement of bread texture and specific volume

The texture of the bread, including hardness and elasticity, were measured by using a texturing apparatus (TA.XT plus, Stable Micro Systems, England) according to the method described by Liu et al. (2018), with some modifications. The cooled bread core was cut into slices of 20 mm^3^, and the test parameters were set as follows: TA4 probe, the pre‐ and post‐test speed of 2 and 1 mm/s, respectively, the test speed of 1 mm/s, the strain of 50%, and the trigger force 5 g. The specific volume of the bread was measured using the rapeseed displacement method described by AACC (2000).

### 2.6 Determination of the content of TPC

The bread was cut into 1‐cm‐thick slices, dried in an oven at 40℃ for 24 h, and then ground into bread powder. Phenols were extracted from the bread flour with 60% ethanol (v/v) at the ratio of 1 g/55 ml at 50℃ for 2 h by shaking (100 rpm), then centrifuged at 10,000 g for 20 min. The supernatant was used for the determination of TPC. The TPC was determined using the Folin–Ciocalteu method described by Singleton and Rossi (1964). TPC is expressed as mg gallic acid equivalents (GAE)/100 g bread weight.

### 2.7 Determination of TCC

According to the methods described by Tang et al. (2015), 0.5 g of the bread powder was extracted with 5 ml ethanol/BHT (100:1 v/w) at 85℃ for 5 min, then mixed with 0.5 ml KOH (80%), and saponified for 10 min, then cooled down, mixed with 3 ml deionized water and 3 ml hexane, centrifuged at 7500 g for 5 min, and the absorbance of the supernatant was measured at 450 and 503 nm. The TCC was calculated as follows:

$$\text{TCC}=4.642\times A_{450}‐3.091\times A_{503}$$

where *A* is the absorbance and TCC is expressed as mg carotene/100 g bread weight.

### 2.8 Determination of digestive characteristics of bread starch

The in vitro digestion characteristics of the bread were measured according to the method described by Huang et al. (2016), with slight modifications. Briefly, 0.2 g bread powder was mixed with phosphate buffer (15 ml, pH 5.2) and equilibrated to 37℃ in a water bath shaker for 20 min. Next, 5 ml of mixed enzymes (290 U/ml porcine pancreas α‐amylase and 15 U/ml amyloglucosidase) was added for enzymatic hydrolysis. At 0, 20, and 120 min, 0.5 ml hydrolytic solution was taken and mixed with 4 ml 80% ethanol for enzyme inactivation. After centrifugation at 4000 g for 10 min, 1 ml of supernatant was taken and mixed with 2 ml 3,5‐dinitrosalicyclic acid (DNS) reagent for the determination of glucose content.

### 2.9 Statistical analysis

Data analysis was conducted using SPSS 20.0 software. All data were measured in triplicate and expressed as mean ± standard deviation (SD). The significant differences between the samples (*p* < .05) were determined using variance and Duncan multiple comparison.

## 3 RESULTS AND DISCUSSION

### 3.1 Determination of the pasting characteristics of potato (sweet potato)‐wheat mixed flour

Table 1 shows that the peak viscosity of mixed flour decreased gradually with the addition of PF and SPF, especially with the latter. Zhou et al. (2013) indicated that starch particles with smaller size formed tighter bonds between molecules, which increased the expansion resistance, thus decreasing the viscosity. Guo et al. (2019) indicated that the particle size of starch of SP was mainly due to the varieties and had no relationship with its color. With the increase in the addition of PF, the breakdown viscosity of the mixed flour increased first and then decreased; however, the increase in the addition of SPF significantly decreased the breakdown viscosity, which indicated that the addition of SPF greatly enhanced the shear resistance and agitation resistance of the flour (Guo et al., 2019). These results were due to the tighter binding among the smaller particle size of starch molecules, which made them difficult to destroy (He et al., 2017).

**TABLE 1:** Effect of adding PF, OFSPF, and PFSPF on the pasting characteristics of mixed flour

Flour added	Addition (%)	Peak viscosity (mPa s)	Trough viscosity (mPa·s)	Breakdown viscosity (mPa·s)	Final viscosity (mPa·s)	Setback viscosity (mPa·s)	Pasting temperature (℃)
PF	0	1813 ± 25^a^	926 ± 11^a^	887 ± 12 ^c^	2183 ± 43^a^	1257 ± 17^a^	83.7 ± 0.17^d^
	10	1754 ± 16^b^	774 ± 22^b^	980 ± 11 ^a^	1723 ± 43^b^	949 ± 8^b^	83.1 ± 0.10^b^
	15	1633 ± 17^c^	695 ± 33^c^	938 ± 10 ^b^	1579 ± 36^c^	884 ± 10^c^	82.5 ± 0.10^d^
	20	1554 ± 29^d^	680 ± 19^cd^	874 ± 14^c^	1519 ± 31^c^	839 ± 9^d^	82.8 ± 0.20^c^
	25	1517 ± 11^e^	666 ± 21^cd^	851 ± 11^d^	1461 ± 17^d^	795 ± 15^e^	82.5 ± 0.10^d^
	30	1447 ± 12^f^	650 ± 18^d^	797 ± 14^e^	1397 ± 37^e^	747 ± 11^f^	82.5 ± 0.10^d^
OFSPF	0	1813 ± 25^a^	926 ± 11^a^	887 ± 12^a^	2183 ± 43^a^	1257 ± 17^a^	83.7 ± 0.17^d^
	10	1071 ± 6^b^	592 ± 19^b^	479 ± 18^b^	1261 ± 23^b^	669 ± 17^b^	84.6 ± 0.17^d^
	15	936 ± 8^c^	502 ± 21^c^	434 ± 21^c^	1021 ± 23^c^	519 ± 9c	84.9 ± 0.26^d^
	20	840 ± 14^d^	447 ± 21^d^	393 ± 13^d^	907 ± 28^d^	460 ± 11^d^	85.3 ± 0.17^c^
	25	736 ± 12^e^	402 ± 15^e^	334 ± 14^e^	789 ± 16^e^	387 ± 10^e^	86.0 ± 0.17^b^
	30	617 ± 8^f^	350 ± 12^f^	267 ± 12^f^	660 ± 17^f^	310 ± 16^f^	86.3 ± 0.17^a^
PFSPF	0	1813 ± 25^a^	926 ± 11^a^	887 ± 12^a^	2183 ± 43^a^	1257 ± 17^a^	83.7 ± 0.17^d^
	10	1016 ± 6^b^	554 ± 11^b^	462 ± 14^b^	1325 ± 16^b^	771 ± 11^b^	85.1 ± 0.10^c^
	15	967 ± 11^c^	537 ± 11^b^	430 ± 14^c^	1259 ± 21^c^	722 ± 11^c^	85.4 ± 0.20^b^
	20	825 ± 10^d^	470 ± 12^c^	355 ± 14^d^	1095 ± 26^d^	625 ± 11^d^	85.9 ± 0.10^a^
	25	719 ± 7^e^	414 ± 10^d^	305 ± 11^e^	941 ± 20^e^	521 ± 11^e^	86.0 ± 0.20^a^
	30	671 ± 20^f^	399 ± 19^d^	272 ± 11^f^	885 ± 12^f^	486 ± 10^f^	86.0 ± 0.20^a^

Note

Different letters in the same column indicate significant differences between samples (*p* < .05).

Abbreviations: OFSPF, orange‐fleshed sweet potato flour; PF, potato flour; PFSPF, purple‐fleshed sweet potato flour.

The addition of PF and SPF reduced the setback viscosity of the flour, suggesting a decrease in the aging of amylose (Guo et al., 2019), among which the effect of OFSPF on the decrease of the aging of amylose was the most obvious. The effect of OFSPF may be due to its lower amylose content (He et al., 2017). Compared with pure WF, the pasting temperature of the flour showed a downward trend after the addition of PF, whereas the effects of SPF were opposite, which may be due to the lower degree of crystallization of starch in PF, resulting in it being easily gelatinized (Chen et al., 2014).

### 3.2 Effect of adding PF and SPF on the thermomechanical characteristics of the dough

Table 2 shows that the addition of PF and PFSPF could increase the water absorption rate of the dough, which may be due to the water‐holding ability of their rich starch and dietary fiber (He et al., 2017; Zhang & Li, 2017). For the OFSPF, with increased addition, the water absorption of the dough increased first, and then decreased, which may be due to the difference in starch and dietary fiber among the varieties, such as starch content and size (Zhang & Li, 2017), as well as the number of hydroxyl groups in the fiber (Rosell et al., 2001). Compared with WF, the addition of PF and SPF reduced the formation and stabilization times of the dough. The increase in the protein‐weakening degree of the dough, which is consistent with the result of He et al. (2017) regarding the effect of the addition of PFSPF on the thermomechanical characteristics of bread dough, suggests that the addition of PF and SPF weakened the dough strength and decreased its stirring endurance (Cao et al., 2017). Zhang et al. (2017), who studied the effect of the addition of PF on the property of dough for making pizza base, also obtained similar results. Owing to the high water absorption ability and low content of gluten protein of PF and SPF, the addition of large amounts of the PF and SPF will cause excessive water absorption of gluten and a decrease in gluten protein content in the mixed flour, thus resulting in a decrease in the gluten strength and stability of the dough. The setback value of the dough decreased with the addition of PF and SPF, indicating that their addition could inhibit starch recrystallization and retrogradation (Cao et al., 2017), which is consistent with the result regarding the setback value of the mixed flour.

**TABLE 2:** Effect of adding PF, OFSPF, and PFSPF on the thermomechanical characteristics of the dough

Flour added	Addition (%)	Water absorption rate (%)	Formation time (min)	Stabilization time (min)	Protein weakening degree (N·m)	Gelatinization (N·m)	Set back value (N·m)
PF	0	63.2 ± 0.26^b^	7.23 ± 0.10^a^	10.05 ± 0.09^a^	0.56 ± 0.07^b^	0.97 ± 0.05^a^	0.80 ± 0.04^a^
	10	68.9 ± 0.26^e^	6.30 ± 0.05^b^	9.17 ± 0.04^b^	0.70 ± 0.06^b^	0.98 ± 0.04^a^	0.51 ± 0.03^b^
	15	72.1 ± 0.20^d^	5.50 ± 0.12^c^	8.03 ± 0.10^c^	0.74 ± 0.07^ab^	0.91 ± 0.04^a^	0.47 ± 0.02^bc^
	20	75.3 ± 0.44^c^	5.20 ± 0.08^d^	7.45 ± 0.08^d^	0.78 ± 0.08^ab^	0.82 ± 0.04^b^	0.47 ± 0.03^bc^
	25	78.5 ± 0.36^b^	5.80 ± 0.06^d^	6.73 ± 0.08^e^	0.81 ± 0.04^ab^	0.71 ± 0.04^c^	0.45 ± 0.03^c^
	30	81.9 ± 0.20^a^	5.50 ± 0.06^e^	6.08 ± 0.09^f^	0.83 ± 0.05^a^	0.59 ± 0.04^d^	0.45 ± 0.03^c^
OFSPF	0	63.2 ± 0.26^b^	7.23 ± 0.10^a^	10.05 ± 0.09^a^	0.56 ± 0.07^b^	0.97 ± 0.05^a^	0.80 ± 0.04^a^
	10	63.7 ± 0.20^a^	5.37 ± 0.06^d^	9.32 ± 0.12^b^	0.70 ± 0.04^a^	0.97 ± 0.05^a^	0.75 ± 0.09^a^
	15	63.8 ± 0.30^a^	5.90 ± 0.01^c^	6.93 ± 0.07^c^	0.73 ± 0.08^a^	0.95 ± 0.02^a^	0.64 ± 0.13^b^
	20	61.6 ± 0.20^c^	5.88 ± 0.06^c^	6.73 ± 0.09^d^	0.78 ± 0.03^a^	0.91 ± 0.04^ab^	0.56 ± 0.19^c^
	25	61.6 ± 0.30^c^	6.78 ± 0.07^b^	6.25 ± 0.07^e^	0.78 ± 0.07^a^	0.88 ± 0.03^c^	0.48 ± 0.26^d^
	30	60.4 ± 0.20^d^	6.67 ± 0.08^b^	5.75 ± 0.09^f^	0.77 ± 0.04^a^	0.90 ± 0.03^ab^	0.41 ± 0.18^e^
PFSPF	0	63.2 ± 0.26^b^	7.23 ± 0.10^a^	10.05 ± 0.09^a^	0.56 ± 0.07^b^	0.97 ± 0.05^a^	0.80 ± 0.04^a^
	10	68.6 ± 0.30^e^	4.80 ± 0.07^bc^	7.82 ± 0.06^b^	0.82 ± 0.04^a^	0.99 ± 0.04^a^	0.53 ± 0.03^b^
	15	71.5 ± 0.20^d^	4.67 ± 0.09^c^	7.23 ± 0.05^c^	0.86 ± 0.03^a^	0.95 ± 0.02^a^	0.48 ± 0.02^c^
	20	74.5 ± 0.20^c^	4.83 ± 0.08^b^	6.77 ± 0.08^d^	0.85 ± 0.03^a^	0.87 ± 0.03^b^	0.47 ± 0.03^cd^
	25	77.5 ± 0.30^b^	1.50 ± 0.07^d^	6.37 ± 0.06^e^	0.86 ± 0.03^a^	0.80 ± 0.02^c^	0.43 ± 0.03^cd^
	30	80.6 ± 0.10^a^	1.45 ± 0.07^d^	6.25 ± 0.07^e^	0.84 ± 0.04^a^	0.72 ± 0.03^d^	0.42 ± 0.02^d^

Note

Different letters in the same column indicate significant differences between samples (*p* < .05).

Abbreviations: OFSPF, orange‐fleshed sweet potato flour; PF, potato flour; PFSPF, purple‐fleshed sweet potato flour.

### 3.3 Effect of the addition of PF and SPF on bread texture

**FIGURE 1:**
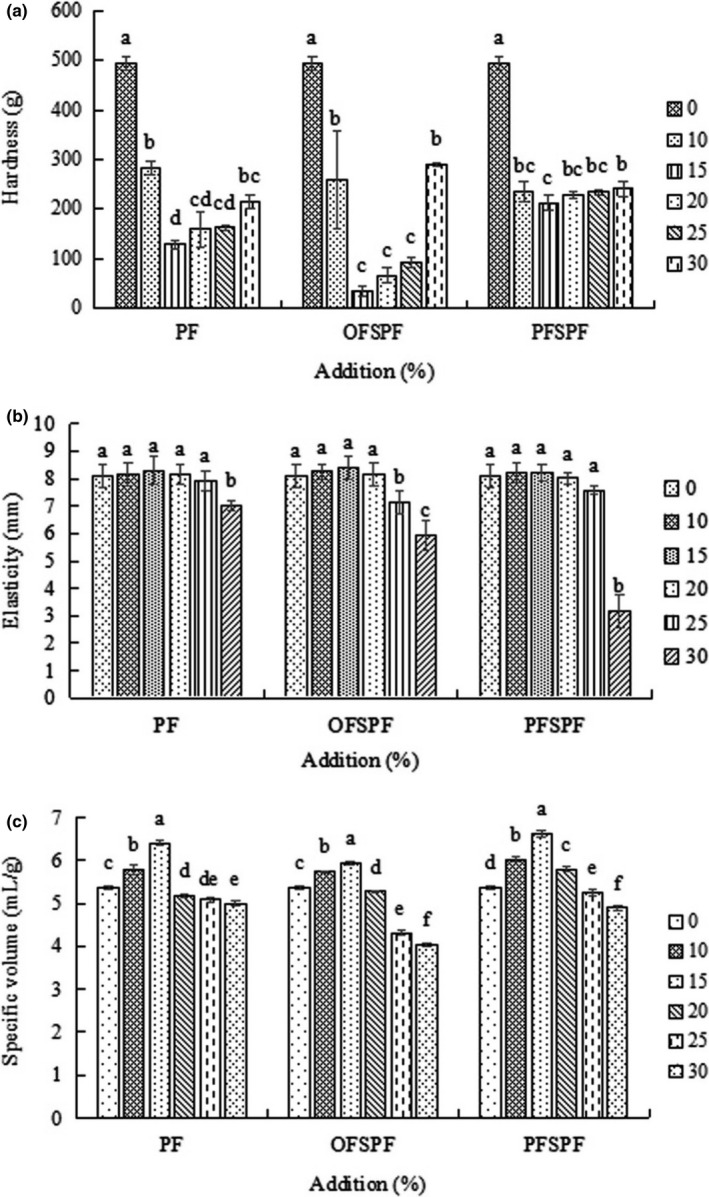
Effect of addition of PF, OFSPF, and PFSPF on the (a) hardness, (b) elasticity, and (c) specific volume of bread (*p* < .05). OFSPF, orange‐fleshed sweet potato flour; PF, potato flour; PFSPF, purple‐fleshed sweet potato flour

Figure 1a shows the change in the hardness of the bread with the increase in the addition of PF and SPF. The hardness of the bread with added PF and SPF is significantly lower than that with no addition (*p* < .05). When the added amount reached 15%, the hardness of the bread reached the lowest. Among them, the hardness of the bread with added OFSPF was the lowest (35.23 ± 0.47 g), and that with PFSPF was the highest. Good‐quality bread means bread with low hardness, as well as high elasticity (Tong et al., 2010). However, with increased addition, the hardness of the bread decreased first, and then increased (*p* < .05), which is consistent with the result of Cao et al. (2019) regarding the effect of substitution with potato pulp for wheat on the quality of steamed bread, indicating that the addition of PF and SPF should be appropriate (15% is the best). There is a lack of gluten protein in potato and sweet potato; therefore, when the addition of PF and SPF increases beyond 15%, the reduced gluten protein content makes it difficult to form a strong three‐dimensional network structure inside the dough to hold the air. This results in bread with a tight tissue structure, thereby increasing the hardness gradually (Cao et al., 2019).

The changes in the elasticity and specific volume of the bread showed opposite trends compared with that in hardness (Figure 1b,c). With the addition of flour, elasticity increased slightly (*p* > .05); when the addition of flour reached 30%, the elasticity decreased significantly, which is consistent with the result of Cao et al. (2019), due to the reduced gluten protein content leading to the reduction in the ability of the dough to keep the gas (Cao et al., 2019; Zhang et al., 2017). When the addition amount was 10%‐15% for SP and OFSPF and 10%‐20% for PFSPF, the specific volume of the bread was higher than that with no addition. When the amount was 15%, the specific volume reached the maximum. Among them, the bread with added PFSPF had the highest specific volume.

Potato and sweet potato are rich in minerals, such as potassium and magnesium, which can promote the growth of yeast and produce more gas. In addition, the potato and sweet potato starch in the gluten network of the dough absorbs more water to swell during gelatinization, resulting in an increase in the specific volume of the bread during baking. However, the lack of gluten protein and the rich dietary fiber and phenolics in PF and SPF caused damage in the gluten network structure and led to the failure of the dough to fully expand during fermentation, which eventually resulted in the decrease in the specific volume of the bread (Han & Koh, 2011; Li et al., 2015).

### 3.4 Effects of PF and SPF addition on TPC and TCC

**FIGURE 2:**
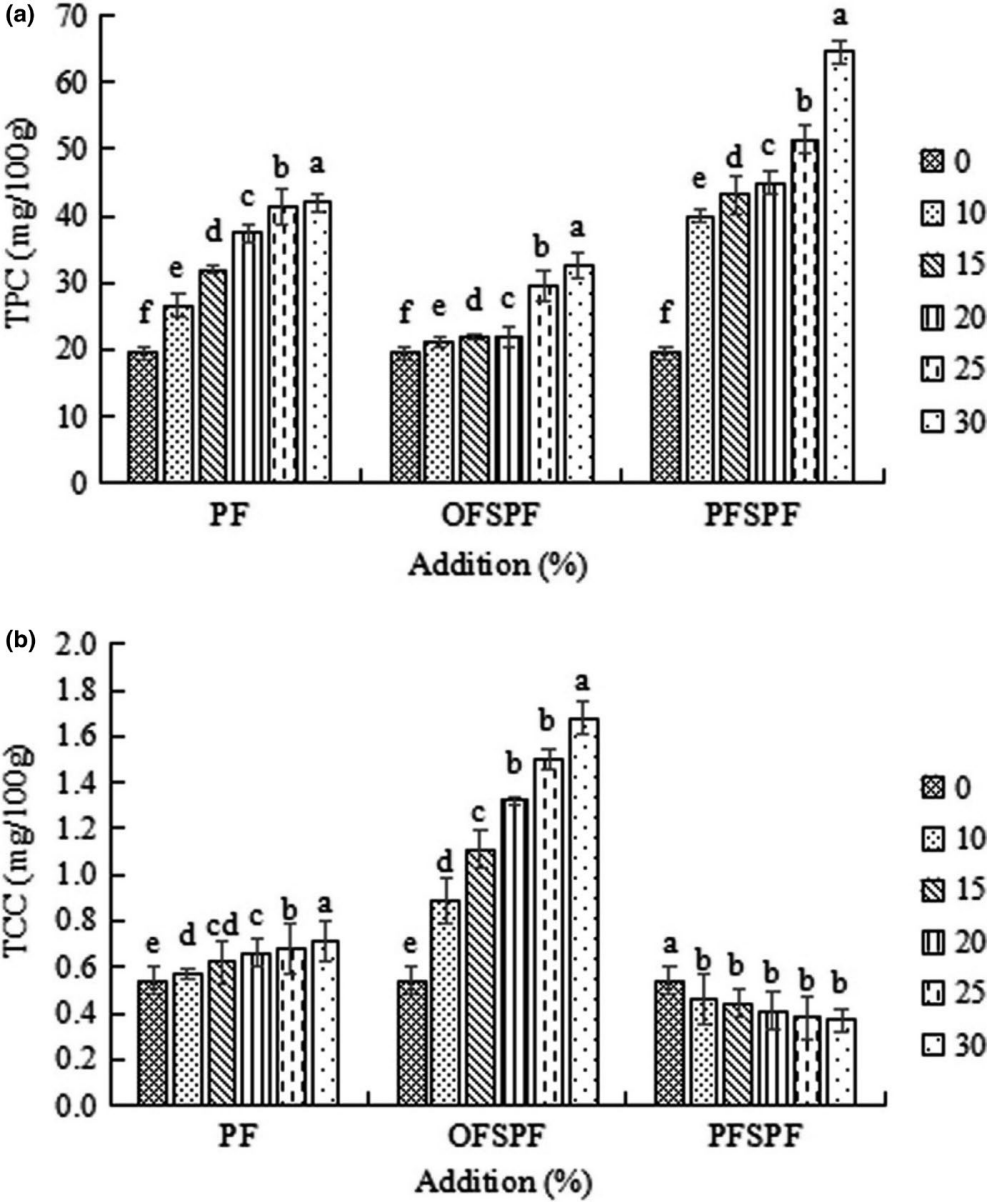
Effect of addition of PF, OFSPF, and PFSPF on (a) TPC and (b) TCC of bread (*p* < .05). OFSPF, orange‐fleshed sweet potato flour; PF, potato flour; PFSPF, purple‐fleshed sweet potato flour; TCC, total carotenoid content; TPC, total polyphenol content

With an increase in the amounts of PF and SPF, the TPC of the bread significantly increased (Figure 2a). Among them, the bread with OFSPF had the highest TCC (Figure 2b), but the lowest TPC, whereas the bread with PFSPF had the highest TPC, but the lowest TCC, indicating the difference in TPC and TCC of PF, OFSPF, and PFSPF. The result of the correlation analysis (Table 4) showed that TPC was negatively correlated with elasticity and specific volume (*p* < .01), which is consistent with the result of Han and Koh (2011) regarding the decrease in a specific volume of bread influenced by adding phenolic acids to the formula. It was reported that phenolics tended to weaken the network of gluten and reduced the specific volume of bread through interaction with the disulfide bond and the thiol group of gluten (Xu et al., 2019). In addition, TCC had the same correlation with the elasticity and specific volume of bread (for the bread with PFSPF, the correlation analysis was not performed considering its low TCC content), but the related study was not reported.

### 3.5 Correlation of TPC, TCC with bread texture, and digestive characteristics of bread starch

With the addition of PF, OFSPF, and PFSPF, the content of rapidly digestible starch (RDS) and slowly digestible starch (SDS) in the bread decreased, while the content of resistant starch (RS) increased (Table 3). Moreover, the bread with SPF had a higher content of RS, lower RDS and SDS contents when compared with those with PF. The correlation analysis for TPC, TCC, and the digestive characteristics of the bread (Table 4) showed that TPC and TCC positively correlated with the RS content and negatively correlated with the RDS and SDS contents, indicating that the polyphenols and carotenoids in the potato and sweet potato improved the digestive characteristics of starch. Rocchetti et al. (2020) found that anthocyanins and procyanidins can change the starch digestibility of sorghum flour in vitro, and have a strong correlation with RS, which is consistent with our results. This may be due to the interaction between polyphenols and starch to form an insoluble complex with high hydrolysis resistance (Moraes et al., 2015). In addition, Kim and Huber (2016) found that starch and β‐carotene formed starch‐β‐carotene‐ordered structures, which were resistant to enzymatic hydrolysis.

**TABLE 3:** Effect of adding PF, OFSPF, and PFSPF on the digestibility of bread

Flour added	Addition (%)	RDS (%)	SDS (%)	RS (%)
PF	0	19.23 ± 0.22^a^	15.46 ± 0.18^a^	66.03 ± 0.12^d^
	10	18.29 ± 0.56^b^	13.01 ± 0.54^b^	68.70 ± 0.83^a^
	15	17.44 ± 0.30^c^	12.23 ± 0.63^bc^	70.33 ± 0.75^b^
	20	16.21 ± 0.28^d^	11.74 ± 0.90^c^	72.06 ± 1.16^c^
	25	14.01 ± 0.40^e^	9.69 ± 0.40^d^	76.31 ± 0.58^c^
	30	12.95 ± 0.48^f^	9.51 ± 1.06^d^	77.54 ± 1.53^d^
OFSPF	0	19.23 ± 0.22^a^	15.46 ± 0.18^a^	66.03 ± 0.12^d^
	10	14.98 ± 0.29^b^	14.01 ± 0.27^b^	71.01 ± 0.56^d^
	15	13.92 ± 0.23^c^	12.56 ± 0.11^c^	73.52 ± 0.35^c^
	20	13.18 ± 0.19^cd^	11.03 ± 0.54^d^	75.78 ± 0.62^b^
	25	13.00 ± 0.89^d^	10.31 ± 0.96^d^	76.69 ± 1.85^b^
	30	10.68 ± 0.21^e^	8.24 ± 0.30^e^	81.09 ± 0.51^a^
PFSPF	0	19.23 ± 0.22^a^	15.46 ± 0.18^a^	66.03 ± 0.12^d^
	10	17.64 ± 0.45^b^	12.46 ± 0.45^c^	69.91 ± 0.90^d^
	15	16.52 ± 0.17^c^	14.49 ± 0.03^b^	68.99 ± 0.19^e^
	20	13.57 ± 0.15^d^	12.00 ± 0.16^c^	74.43 ± 0.29^c^
	25	10.18 ± 0.17^e^	11.21 ± 0.47^d^	78.61 ± 0.63^b^
	30	9.47 ± 0.49^f^	6.93 ± 0.05^e^	83.61 ± 0.48^a^

Note

Different letters in the same column indicate significant differences between samples (*p* < .05).

Abbreviations: OFSPF, orange‐fleshed sweet potato flour; PF, potato flour; PFSPF, purple‐fleshed sweet potato flour; RDS, rapid digestible starch; RS, resistant starch; SDS, slowly digestible starch.

**TABLE 4:** Correlation analysis between TPC, TCC, and bread texture, starch digestibility

		Texture characteristics			Digestive characteristics		
		Hardness	Elasticity	Specific volume	RDS	SDS	RS
TPC	PWB	−0.019	−0.740**	−0.687**	−0.951**	−0.883**	0.938**
	OFSPWB	−0.047	−0.718**	−0.888**	−0.838**	−0.931**	0.776**
	PFSPWB	0.358	−0.934**	−0.903**	−0.859**	−0.976**	0.960**
TCC	PWB	−0.327	−0.574*	−0.722**	−0.940**	−0.859**	0.922**
	OFSPWB	0.124	−0.821**	−0.928**	−0.912**	−0.968**	0.866**

Abbreviations: OFSPWB, orange‐fleshed sweet potato‐wheat bread; PFSPWB, purple‐fleshed sweet potato‐wheat bread; PWB, potato‐wheat bread; RDS, rapid digestible starch; RS, resistant starch; SDS, slowly digestible starch; TCC, total carotenoid content; TPC, total polyphenol content; * Significant correlation at 0.05 level; ** Significant correlation at 0.01 level.

## 4 CONCLUSION

In this study, the addition of 10%–30% PF, OFSPF, and PFSPF to WF influenced the pasting properties of flour, thermomechanical properties of dough, texture, and TPC, TCC, as well as starch digestibility of bread. Except for the pasting temperature, with the addition of PF and SPF, the peak, breakdown, and setback viscosity decreased. However, the extent of the decrease depended on the flour variety; this was most evident for the flour with added SPF in the peak and breakdown viscosity, and for the flour with added OFSPF in the setback viscosity. The addition of PF and SPF reduced the formation and stabilization time of the dough, as well as the hardness of the bread. However, the specific volume of bread depended on the amount of different flour added. When the proportion was 10%–15% for SP and OFSPF and 10%–20% for PFSPF, the specific volume of the bread was higher than that of WF bread. When the proportion was 15%, the hardness was the lowest while the specific volume was the highest. The bread with added OFSPF and PFSPF had the lowest hardness and highest specific volume, respectively. The TPC and TCC in the bread depended on the flour variety added, and were negatively correlated with the specific volume, elasticity, and RDS content and positively correlated with the RS content.

## CONFLICT OF INTEREST

None.

## AUTHOR CONTRIBUTIONS

**Hongwei Meng:** dataCuration (equal); formalAnalysis (equal); investigation (equal); methodology (equal); validation (equal); visualization (equal); writingOriginalDraft (equal). **Chong Xu:** dataCuration (equal); formalAnalysis (equal); methodology (equal); resources (equal); visualization (equal). **Meiying Wu:** dataCuration (equal); formalAnalysis (equal); visualization (equal). **Ying Feng:** conceptualization (equal); formalAnalysis (equal); fundingAcquisition (equal); methodology (equal); projectAdministration (equal); resources (equal); supervision (equal); validation (equal); writingReviewEditing (equal).

## Data Availability

The data that support the findings of this study are available from the corresponding author upon reasonable request.
